# Ectopic bile duct concomitant with gastric ulcer hemorrhage: report of a case

**DOI:** 10.1186/s40792-024-01867-0

**Published:** 2024-03-15

**Authors:** Yuiko Nagasawa, Masayuki Ohta, Yuki Shitomi, Hiroshi Satoh, Masanori Aramaki

**Affiliations:** Department of Surgery, Oita Oka Hospital, 3-7-11 Nishitsurusaki, Oita, Japan

**Keywords:** Ectopic bile duct, Gastric ulcer, Hemorrhage, Gastric ulcer hemorrhage, Stomach

## Abstract

**Background:**

An ectopic bile duct opening into the stomach is a rare congenital anomaly of the biliary system, and thus, there are few case reports with gastric ulcer hemorrhage. Herein, we presented a case of ectopic bile duct concomitant with gastric ulcer hemorrhage.

**Case presentation:**

A 75-year-old woman was referred to our hospital because she repeatedly vomited blood and had melena. Endoscopic hemostasis was attempted for hemorrhage from a gastric ulcer located on the anterior wall of the antrum. However, the bleeding was difficult to stop, and a laparoscopic distal gastrectomy was performed. Her postoperative course was uneventful. Pathological examination revealed that the bleeding point was an ectopic bile duct. In retrospect, an annual endoscopy performed at her family clinic had revealed a bulge in the same portion of the stomach. Exposure to bile acids from an ectopic bile duct opening can cause gastric mucosal damage and ulceration.

**Conclusions:**

Ectopic bile ducts opening into the stomach can cause gastric ulcer and hemorrhage. Hemorrhage from a submucosal ridge with ulcer in the stomach may be rarely related to the presence of ectopic bile ducts.

## Introduction

An ectopic bile duct opening into the stomach is a rare congenital anomaly. Some cases are asymptomatic and undetected, and the prevalence is unknown. As screening modalities have evolved, two cases have been discovered incidentally [1, 2]. These cases were reported from Japan, probably because of the Japanese culture of undergoing regular check-ups for stomach and gastric cancer even if there are no symptoms. Many other published reports indicate duplication of the common bile duct opening into the stomach [1–18]. Herein, we report a case of hemorrhagic gastric ulcer, which was diagnosed as being due to an ectopic bile duct by postoperative pathological examination.

## Case report

**Fig. 1:**
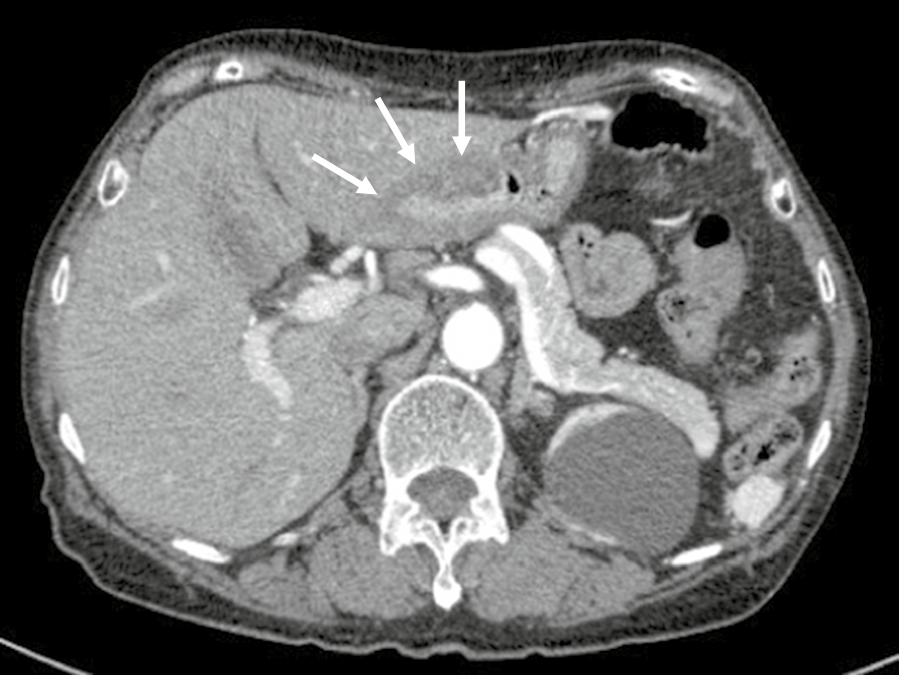
Contrast-enhanced computed tomography image. Increased wall thickness of the gastric antrum was observed (arrows)

A 75-year-old woman had received treatment for herpes zoster on her trunk in a family clinic for 1 week. Thereafter, she repeatedly began to vomit blood and had melena and was referred to our hospital. She had undergone thyroidectomy for thyroid adenomas 20 years ago and had a history of hypertension and cerebral hemorrhage 10 years ago. However, she had no history of abdominal surgery. On presentation, her blood pressure was 116/69 mmHg, heart rate was 78/min, and transcutaneous oxygen saturation was 96%. A blood test revealed anemia (red blood cells 331 × 10^4^/μL and hemoglobin 9.2 g/dL) but normal levels of white blood cells (6800/μL), platelets (21.2 × 10^4^/μL), prothrombin activity (101%), and tumor markers (carcinoembryonic antigen and cancer antigen 19-9). Contrast-enhanced computed tomography showed a thickened wall in the antrum of the stomach (Fig. 1). There was no lymph node swelling around her stomach, but pulmonary nodules were suggestive of tuberculosis, primary lung cancer, or metastatic lung tumor. Computed tomography also showed gallbladder stones, and she had a previous history of colic pain. Because her T-SPOT test was positive, she was isolated until tuberculosis could be ruled out.

**Fig. 2:**
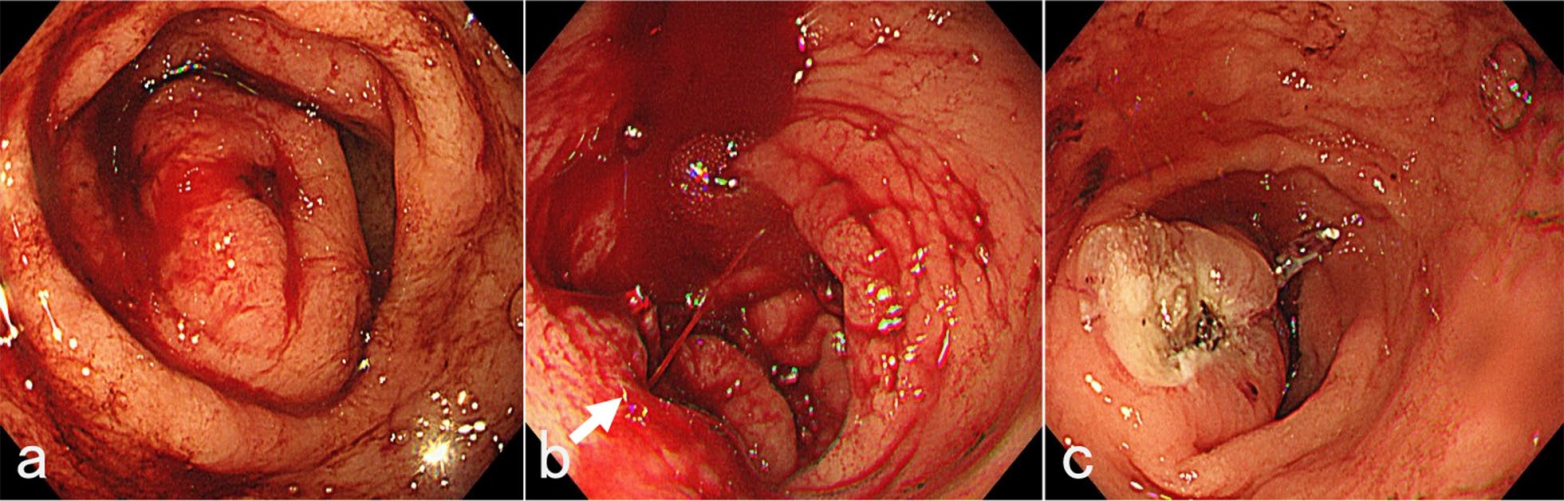
Emergency endoscopic hemostasis. **a** A submucosal ridge with hemorrhage was found on the anterior wall of the gastric antrum. **b** Eruptive bleeding was observed from the center of the ulcer (arrow). **c** Temporary hemostatic findings after electrocoagulation

Endoscopic hemostasis was urgently attempted to stop the bleeding. Upper gastrointestinal endoscopy revealed a submucosal ridge on the anterior wall of the gastric antrum and eruptive bleeding from a central ulcer (Fig. 2a, b). There were no atrophic changes in the gastric mucosa. Her gastric tissue was fragile, which made it difficult to stop the bleeding by clipping. Finally, the bleeding was temporarily stopped by electrocoagulation (Fig. 2c). However, after a blood transfusion, her anemia worsened and melena persisted. Surgery was considered necessary to control the gastric bleeding. Imaging findings suggested the presence of a small submucosal tumor such as a gastrointestinal stromal tumor. Based on the lesion localization, partial gastrectomy was considered to cause postoperative gastric deformity and stenosis, and therefore, laparoscopic distal gastrectomy was chosen in this case. In addition, laparoscopic cholecystectomy was also proposed for treatment of the gallbladder stones.

**Fig. 3:**
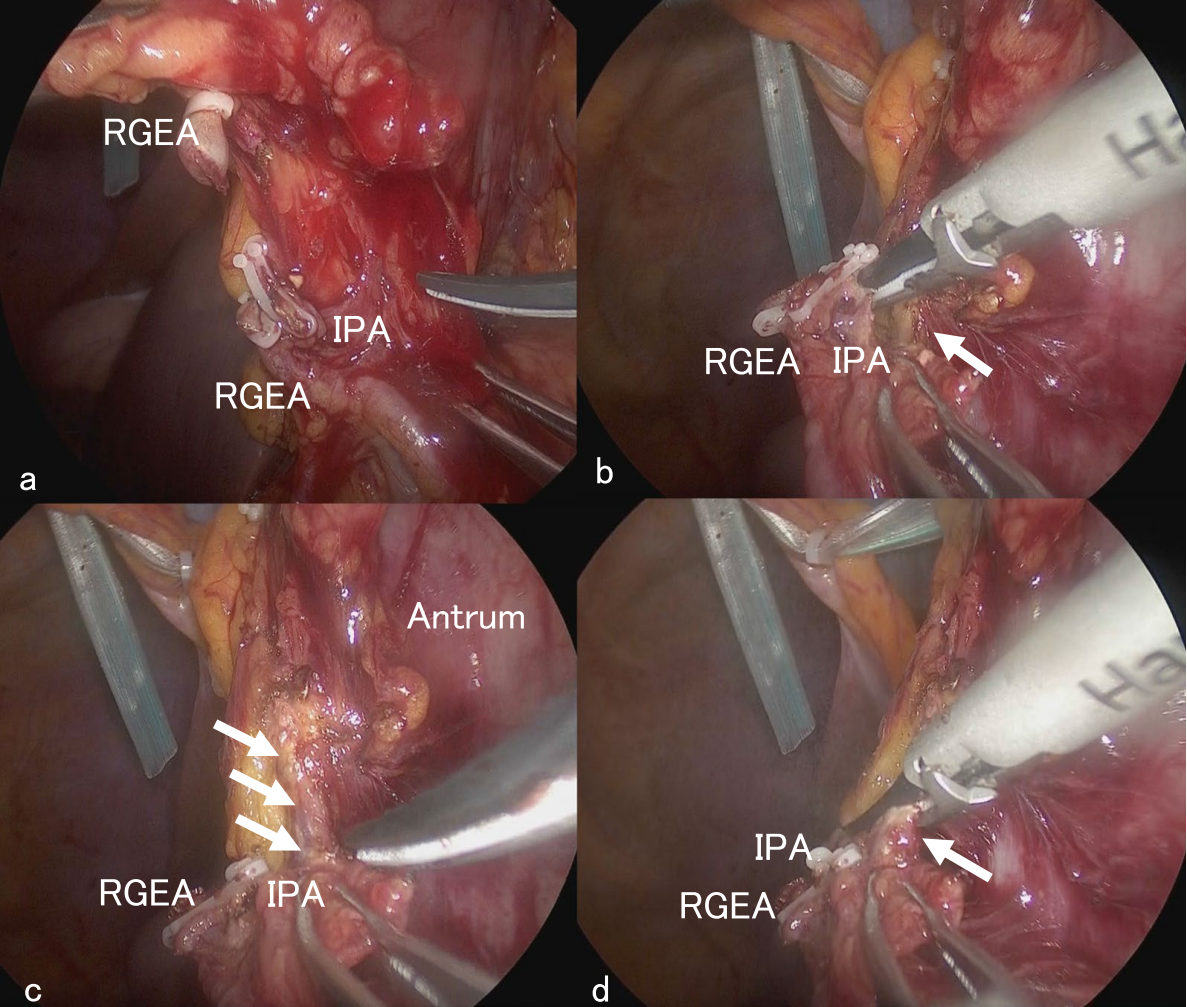
Video image during laparoscopic distal gastrectomy. A thin white cord (arrows) that connected to the antrum from the dorsal side of the stomach was recognized after clipping and division of the right gastroepiploic artery (RGEA) and infrapyloric artery (IPA). **a** Just after division of the RGEA and IPA. **b** A thin white cord (arrow) appeared during dissection of fat tissue. **c** The thin white cord (arrows) was clearly recognized after removal of fat tissue. **d** During dissection of the thin cord (arrow)

**Fig. 4:**
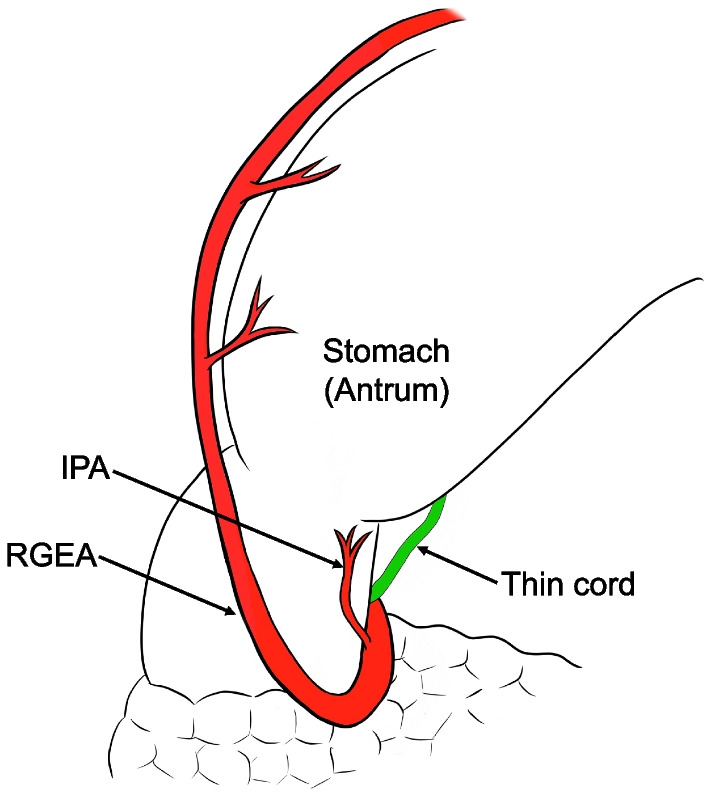
Anatomical features of the thin cord, stomach and vessels. RGEA: right gastroepiploic artery, IPA: infrapyloric artery

**Fig. 5:**
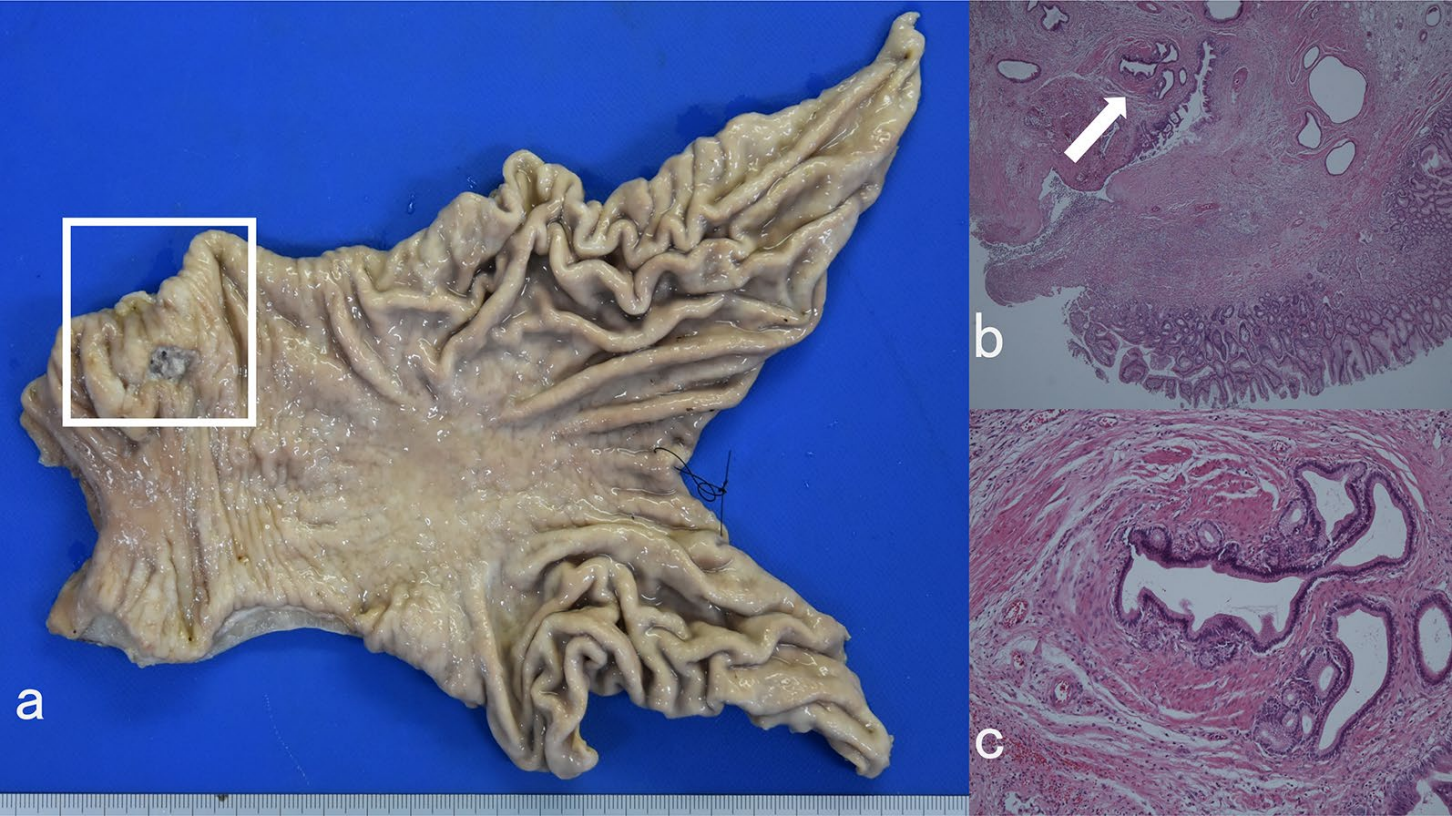
Pathological findings. The area surrounded by the square was coagulated and had a structure closely resembling the papilla of the duodenum (**a** Macroscopic image, **b** Weak magnification (arrow), hematoxylin and eosin (HE) staining, × 100, **c** High magnification, HE staining, × 400). A glandular structure similar to the bile duct was observed. The pathologist diagnosed the tissue at the bed of the gastric ulcer as a bile duct opening

**Fig. 6:**
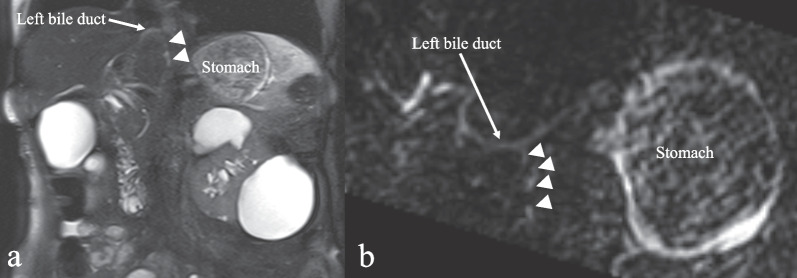
Magnetic resonance imaging. A variant bile duct originating from the left hepatic duct and proceeding to the stomach (arrow heads) was recognized 1 month after the operation (**a** T2-weighted image, **b** diffusion-weighted image)

Under laparoscopy, there were few intraperitoneal adhesions and reduced visceral fat. A thin white cord was present that connected to the lesser curvature of the antrum from the hepatoduodenal ligament (Figs. 3 and 4). The cord was separated by laparoscopic coagulation shears (Harmonic Scalpel, Ethicon Endo-Surgery, Cincinnati, OH, USA). Laparoscopic distal gastrectomy comprising 2/3 stomach resection with D1 lymph node dissection, Roux-en-Y reconstruction, and no preservation of the vagus nerve and laparoscopic cholecystectomy were performed safely. The stomach specimen showed the ulcer area to be soft, and the mass was not palpable. Although it was unknown whether sealing of the cord by laparoscopic coagulation shears was adequate, her postoperative course was uneventful, and she was discharged 14 days after the operation. On pathological examination, no tumor cells were found, and the same structure as a duodenal papilla was pointed out in the raised part of the anterior wall of the gastric antrum (Fig. 5). The thin white cord was connected to this structure posteriorly. Magnetic resonance imaging performed 1 month after the operation revealed a variant bile duct originating from the left hepatic duct and proceeding to the stomach (Fig. 6). Therefore, the ectopic bile duct was not included in the Goor and Ebert classification of double bile ducts [19], and was classified as type IIIb by Saito et al. [20]. In retrospect, an annual endoscopic examination at her family clinic had shown a bulge in the same portion of the antrum. She has been free of complications for 1 year after the operation.

## Discussion

The hepatic fossa is divided into the pars hepatica that forms the hepatic cell cord and hepatic canal and the pars cystica that forms the gall bladder and bile duct, and the common bile duct develops from the common part during the embryonic period. Early separation of the hepatic fossa causes union failure and forms two bile ducts. In addition, the duct ostium opens into the stomach if the accessory bile duct is formed before the stomach and duodenum separate, and into the duodenum if the accessory bile duct is formed after the separation [21]. The drainage route is determined by the time lag between the separation of the stomach and duodenum, with the duodenum as the most common opening, followed by the stomach and pancreatic duct [21]. Goor and Ebert classified the morphological features of double bile ducts into four types in 1972 with double drainage to the duodenum [19]. In Japan, the classification by Saito et al., which is based on the Goor and Ebert classification, is generally used [20]. Duplicated bile ducts occur embryologically during nonunion of the hepatic diverticulum. The bile ducts run through the hepatoduodenal ligament and lesser omentum and open on the side of the lesser curvature when opening into the stomach [22].

To our knowledge, there has been only one reported case of repeated ulcers at the bile duct opening of the stomach [23]. In that case, the opening of the bile duct communicated with the common bile duct. Bile acid has been considered pathogenesis of gastric mucosal damage and ulceration for long time [24]. A high concentration of gastric bile acid has been recognized in patients with gastric ulcer but not in those with duodenal ulcer [25, 26]. Also, bile acid promotes gastric intestinal metaplasia, which is considered a precancerous lesion of gastric cancer [27, 28]. In fact, gastric cancer at an ectopic bile duct opening into the stomach has also been reported [12]. Therefore, exposure to bile acids from an ectopic bile duct opening can cause gastric mucosal damage, ulceration, intestinal metaplasia and cancer. In our case, we found a bile duct opening in the stomach that was similar to the papilla of the duodenum pathologically. Therefore, this appears to be the first case report of an ectopic bile duct concomitant with gastric ulcer hemorrhage. However, this patient had never experienced gastric ulcer and/or ulcer bleeding for more than 70 years until this episode. Just before the hematemesis and melena began, she had received treatment for herpes zoster on her trunk, and the related pain, stress, and medication may also have influenced formation of gastric ulcer.

During the operation, we found a thin cord connecting to the lesser curvature of the antrum from the hepatoduodenal ligament. However, we did not confirm whether the cord communicated with the bile duct because at that time, we did not know that an ectopic bile duct in the stomach could cause gastric ulcer and hemorrhage. After the operation, we found a variant bile duct originating from the left hepatic duct and proceeding to the stomach on magnetic resonance imaging, as was reported previously [18]. Understandably, we could not confirm communication of the variant bile duct into the stomach due to the patient’s post-gastric resection status.

In the present study, differential diagnoses of submucosal protuberances that cause gastric bleeding can include ectopic pancreas, double stomach, gastric hamartoma, hamartomatous inverted polyp, gastrointestinal stromal tumor, leiomyoma, glomus tumor, neuroendocrine tumor, hemangioma, lipoma, lymphangioma, and metastasis and invasion of malignant tumors in other organs. However, we ruled out these lesions by pathological examination. Hamartomatous inverted polyp was suspected due to the presence of ductal structures and smooth muscle tissue in the submucosa of stomach [29]. However, there was no cyst formation, and we ultimately ruled it out.

This study has some limitations. This is a retrospective study, and we did not confirm whether the cord at the dorsal wall communicated with the bile duct. In addition, the paraffin specimen block had already been discarded, and unfortunately, additional immunohistochemical staining could not be performed.

## Conclusions

An ectopic bile duct opening into the stomach can cause gastric ulcer and hemorrhage. Hemorrhage from a submucosal ridge with ulcer in the stomach may be rarely related to an ectopic bile duct.

## Data Availability

The data that support the findings in this study are available from the corresponding author upon reasonable request.

## Abbreviation

HE: Hematoxylin and eosin

## References

[1] Arase Y, Deguchi R, Tsukune Y, Dekiden M, Shiraishi K, Ogimi T (2016). Double common bile duct with ectopic drainage into the stomach found in asymptomatic. Tokai J Exp Clin Med.

[2] Amano Y, Takahashi M, Oishi T, Kumazaki T (2002). MR cholangiopancreatography of double common bile duct with ectopic drainage into stomach. J Comput Assist Tomogr.

[3] Bernard P, Le Borgne J, Dupas B, Kohnen-Shari N, Raoult S, Hamel A (2001). Double common bile duct with ectopic drainage into the stomach. Case report and review of the literature. Surg Radiol Anat.

[4] Park JI, Oh SH (2015). Double common bile duct with an ectopic drainage into the stomach. Ann Surg Treat Res.

[5] Sezgın O, Altintaş E, Uçbılek E (2010). Ectopic opening of the common bile duct into the stomach. Turk J Gastroenterol.

[6] Horsmans Y, De Grez T, Lefebvre V, Witterwulghe M (1996). Double common bile duct with ectopic drainage of the left lobe into the stomach. Case report and review of the literature. Acta Gastroenterol Belg.

[7] Quintana EV, Labat R (1974). Ectopic drainage of the common bile duct. Ann Surg.

[8] Balbinot RA, Gobbato A, Balbinot SS, Mendonça L, Tefilli N, Lain VV (2004). Double bile duct with ectopic drainage into stomach. Gastrointest Endosc.

[9] Kanematsu M, Imaeda T, Seki M, Goto H, Doi H, Shimokawa K (1992). Accessory bile duct draining into the stomach: case report and review. Gastrointest Radiol.

[10] Meng W, Yue P, Bai B, Zhou W, Li X (2017). Narrow band imaging helps identify the ectopic opening of the common bile duct during endoscopic retrograde cholangiopancreatography. Turk J Gastroenterol.

[11] Hekimoglu K, Ustundag Y, Saritas U (2008). Ectopic openings of the bilio-pancreatic ducts in the stomach in an elderly case presenting with choledocholithiasis and acute cholangitis. J Gastrointestin Liver Dis.

[12] Kondo K, Yokoyama I, Yokoyama Y, Harada A, Nagayo T (1986). Two cases of gastric cancer-bearing double choledochus with ectopic drainage into the stomach. Cancer.

[13] Guerra I, Rábago LR, Bermejo F, Quintanilla E, García-Garzón S (2009). Ectopic papilla of Vater in the pylorus. World J Gastroenterol.

[14] Katsinelos P, Papaziogas B, Paraskevas G, Chatzimavroudis G, Koutelidakis J, Katsinelos T (2007). Ectopic papilla of vater in the stomach, blind antrum with aberrant pyloric opening, and congenital gastric diverticula: an unreported association. Surg Laparosc Endosc Percutan Tech.

[15] Nasseri-Moghaddam S, Nokhbeh-Zaeem H, Soroush Z, Sheybani SB, Mazloum M (2011). Ectopic location of the ampulla of vater within the pyloric channel. Middle East J Dig Dis.

[16] Sieber WK, Wiener ES, Chang J (1980). Double choledochus with ectopic drainage into the stomach-a rare congenital anomaly of the biliary ductal system. J Pediatr Surg.

[17] Jain M, Rai GP, Pokharna RK, Nepalia S, Ashdhir P (2015). Incidentally detected ectopic ampulla of vater in the antrum in a patient of colonic tuberculosis. Trop Gastroenterol.

[18] Guan J, Zhang L, Chu JP, Lin SC, Li ZP (2015). Congenital left intrahepatic bile duct draining into gastric wall mimicking biliary reflux gastritis. World J Gastroenterol.

[19] Goor DA, Ebert PA (1972). Anomalies of the biliary tree. Report of a repair of an accessory bile duct and review of the literature. Arch Surg.

[20] Saito N, Nakano A, Arase M, Hiraoka T (1988). A case of duplication of the common bile duct with anomaly of the intrahepatic bile duct (in Japanese). Nihon Geka Gakkai Zasshi.

[21] Boyden EA (1933). The problem of the double ducts choledochus (an interpretation of an accessory bile duct found attached to the parts superior of the duodenum). Anat Rec.

[22] Enomoto K, Nishikawa Y, Nakanuma Y (2017). Development and anatomy of the bile duct. Pathology of the bile duct.

[23] Bauer K, Keller C (2017). Recurrent gastric ulcer and cholangitis caused by ectopic drainage of bile duct into the stomach. GMS Interdiscip Plast Reconstr Surg DGPW.

[24] Duplessis DJ (1965). Pathogenesis of gastric ulceration. Lancet.

[25] Gotthard R, Bodemar G, Tjadermo M, Tobiasson P, Walan A (1985). High gastric acid concentration in prepyloric ulcer patients. Scand J Gastroenterol.

[26] Rydning A, Berstad A (1985). Intragastric bile acid concentrations in healthy subjects and in patients with gastric and duodenal ulcer and influence of fiber-enriched wheat bran in patients with gastric ulcer. Scand J Gastroenterol.

[27] Qu X, Shi Y (2022). Bile reflux and bile acids in the progression of gastric intestinal metaplasia. Chin Med J (Engl).

[28] Wang M, Lou W, Xue Z (2023). The role of bile acid in intestinal metaplasia. Front Physiol.

[29] Tatsuta M, Okuda S, Tamura H, Taniguchi H (1980). Gastric hamartomatous polyps in the absence of familial polyposis coli. Cancer.

