# Correction: Ectopic bile duct concomitant with gastric ulcer hemorrhage: report of a case

**DOI:** 10.1186/s40792-024-01876-z

**Published:** 2024-04-07

**Authors:** Yuiko Nagasawa, Masayuki Ohta, Yuki Shitomi, Hiroshi Satoh, Masanori Aramaki

**Affiliations:** Department of Surgery, Oita Oka Hospital, 3-7-11 Nishitsurusaki, Oita, Japan

**Correction: Surgical Case Reports (2024) 10:63** 10.1186/s40792-024-01867-0

Following publication of the original article [1], the authors reported a mistake in Fig. 4. Due to a typesetting error, the “Stomach (Antrum)” was blurred in Fig. 4.

The original Fig. 4 was:
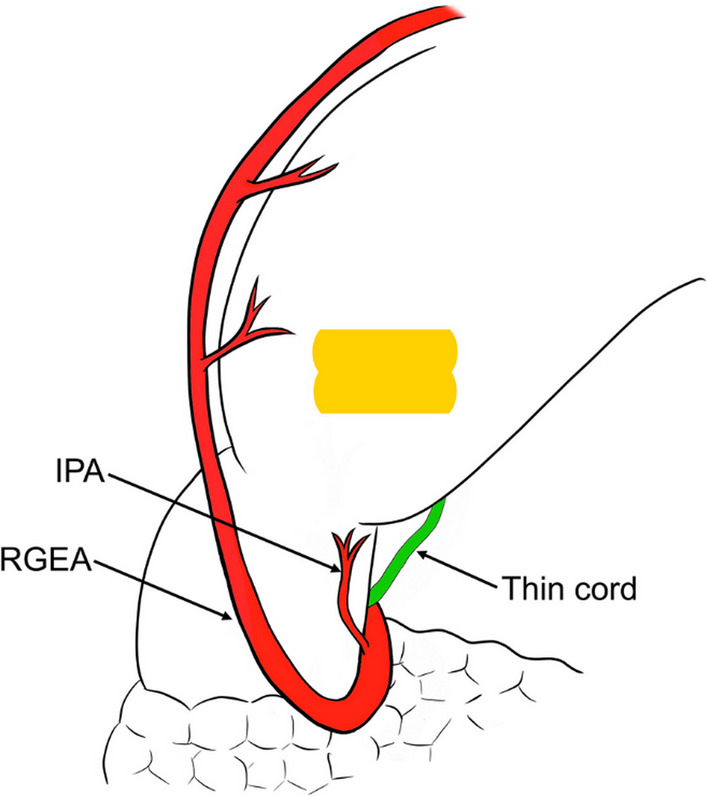


The correct Fig. 4 should read:
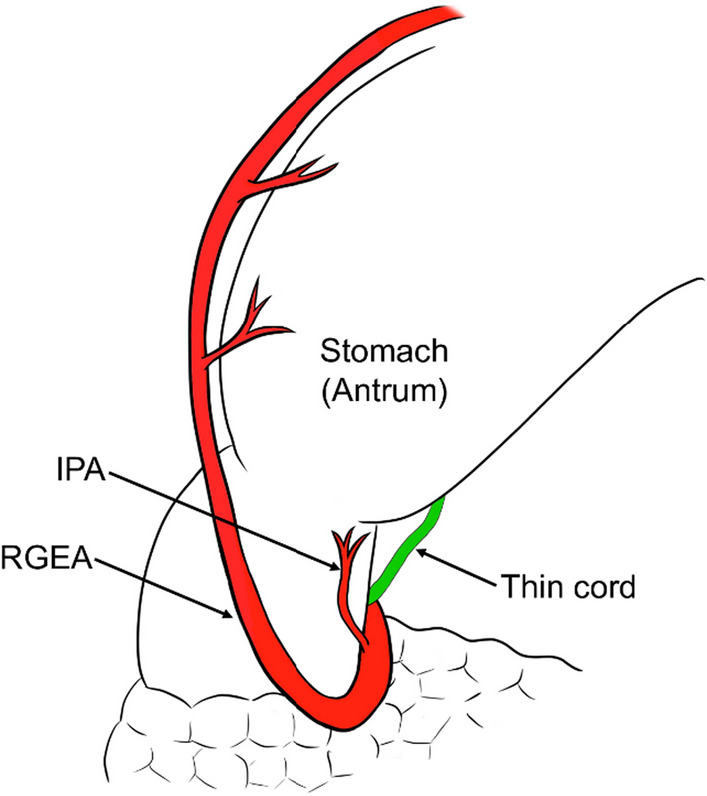


The original article [1] has been updated.
